# Engineering exosomes to reshape the immune microenvironment in breast cancer: Molecular insights and therapeutic opportunities

**DOI:** 10.1002/ctm2.1645

**Published:** 2024-04-04

**Authors:** Jilong Cao, Gang Lv, Fang Wei

**Affiliations:** ^1^ Party Affairs and Administration Office the Fourth Affiliated Hospital of China Medical University Shenyang P. R. China; ^2^ Department of Thyroid and Breast Surgery Chaohu Hospital of Anhui Medical University Chaohu P. R. China; ^3^ Department of General Surgery the Fourth Affiliated Hospital of China Medical University Shenyang P. R. China

**Keywords:** breast cancer, engineered exosomes, immune microenvironment, immunomodulation, immunotherapy

## Abstract

**Background:**

Breast cancer remains a global health challenge, necessitating innovative therapeutic approaches. Immunomodulation and immunotherapy have emerged as promising strategies for breast cancer treatment. Engineered exosomes are the sort of exosomes modified with surface decoration and internal therapeutic molecules. Through suitable modifications, engineered exosomes exhibit the capability to overcome the limitations associated with traditional therapeutic approaches. This ability opens up novel avenues for the development of more effective, personalized, and minimally invasive interventions.

**Main body:**

In this comprehensive review, we explore the molecular insights and therapeutic potential of engineered exosomes in breast cancer. We discuss the strategies employed for exosome engineering and delve into their molecular mechanisms in reshaping the immune microenvironment of breast cancer.

**Conclusions:**

By elucidating the contribution of engineered exosomes to breast cancer immunomodulation, this review underscores the transformative potential of this emerging field for improving breast cancer therapy.

**Highlights:**

1. Surface modification of exosomes can improve the targeting specificity.
2. The engineered exosome‐loaded immunomodulatory cargo regulates the tumour immune microenvironment.
3. Engineered exosomes are involved in the immune regulation of breast cancer.

## Graphical abstract

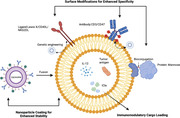

## 1 INTRODUCTION

Breast cancer (BC) represents a significant global health concern, accounting for a substantial portion of cancer‐related deaths worldwide.^1^, ^2^ Conventional therapies, including surgery, radiation and chemotherapy, have demonstrated limited success in advanced‐stage disease.^3^ Recent advances in cancer immunotherapy have opened new avenues for more targeted and effective therapeutic strategies.^4^ At present, BC immunotherapy encompasses various strategies, including immune checkpoint blockade, tumour vaccines, chimeric antigen receptor T (CAR‐T) cell therapy and several other innovative approaches.^5^ However, the road to effective immunotherapy is fraught with complexities, necessitating a nuanced understanding of the immune microenvironment within the BC milieu.^6^

Exosomes, small extracellular vesicles with diameters ranging from 30 to 150 nm, have garnered significant attention for their potential applications in immunotherapy.^7^ Originating from intracellular multivesicular bodies, exosomes are enveloped by the cell membrane before being released into the extracellular environments.^8^ Packed with various biomolecules, including proteins, nucleic acids, lipids and other cellular components, exosomes play a crucial role in intercellular communication.^9^, ^10^ The physiological mechanism of exosomes involves their function as carriers of information, influencing immune responses and cell‐to‐cell communication through the transfer of specific biomolecules.^11^ This includes the regulation of T cells, B cells and antigen‐presenting cells, impacting the intensity and direction of immune responses. Furthermore, the nucleic acid molecules within exosomes, particularly microRNA (miRNA) and messenger RNA, have the ability to modulate gene expression in recipient cells, thereby affecting the activity and function of immune cells.^12^

In recent years, the concept of engineering exosomes has emerged as a transformative approach to harness and enhance their therapeutic potential. Engineered exosomes involve the manipulation of these vesicles to achieve specific modifications in their cargo or surface properties. This engineering process allows researchers to tailor exosomes for targeted drug delivery, diagnostic purposes, or modulation of specific cellular functions.^13^ The modification of exosomes can be achieved through various techniques, including genetic engineering of parent cells, direct exosome loading, or surface modifications. These tailored exosomes can then be utilized in a range of applications, from delivering therapeutic payloads to specific tissues to serving as diagnostic biomarkers or even as vehicles for gene therapy.^14^ The ability to precisely engineer exosomes opens up new avenues for addressing the limitations of traditional therapeutic approaches and enables the development of more effective, personalized and minimally invasive interventions.^15^ Hence, the primary focus in utilizing exosomes for antitumour immunotherapy lies in the exploration of engineered exosomes obtained through various processing methods. In this comprehensive review, we explore the molecular insights and therapeutic potential of engineered exosomes in BC. We discuss the strategies employed for exosome engineering and delve into their molecular mechanisms in reshaping the immune microenvironment of BC. By elucidating the contribution of engineered exosomes to BC immunomodulation, this review underscores the transformative potential of this emerging field for improving BC therapy.

## 2 EXOSOME BIOLOGY AND BIOGENESIS

Exosomes, small extracellular vesicles with a diameter of 30–150 nm, have emerged as potent mediators of intercellular communication.^16^ Their role extends beyond cellular waste disposal, positioning them as crucial players in physiological and pathological processes.^17^

**FIGURE 1:**
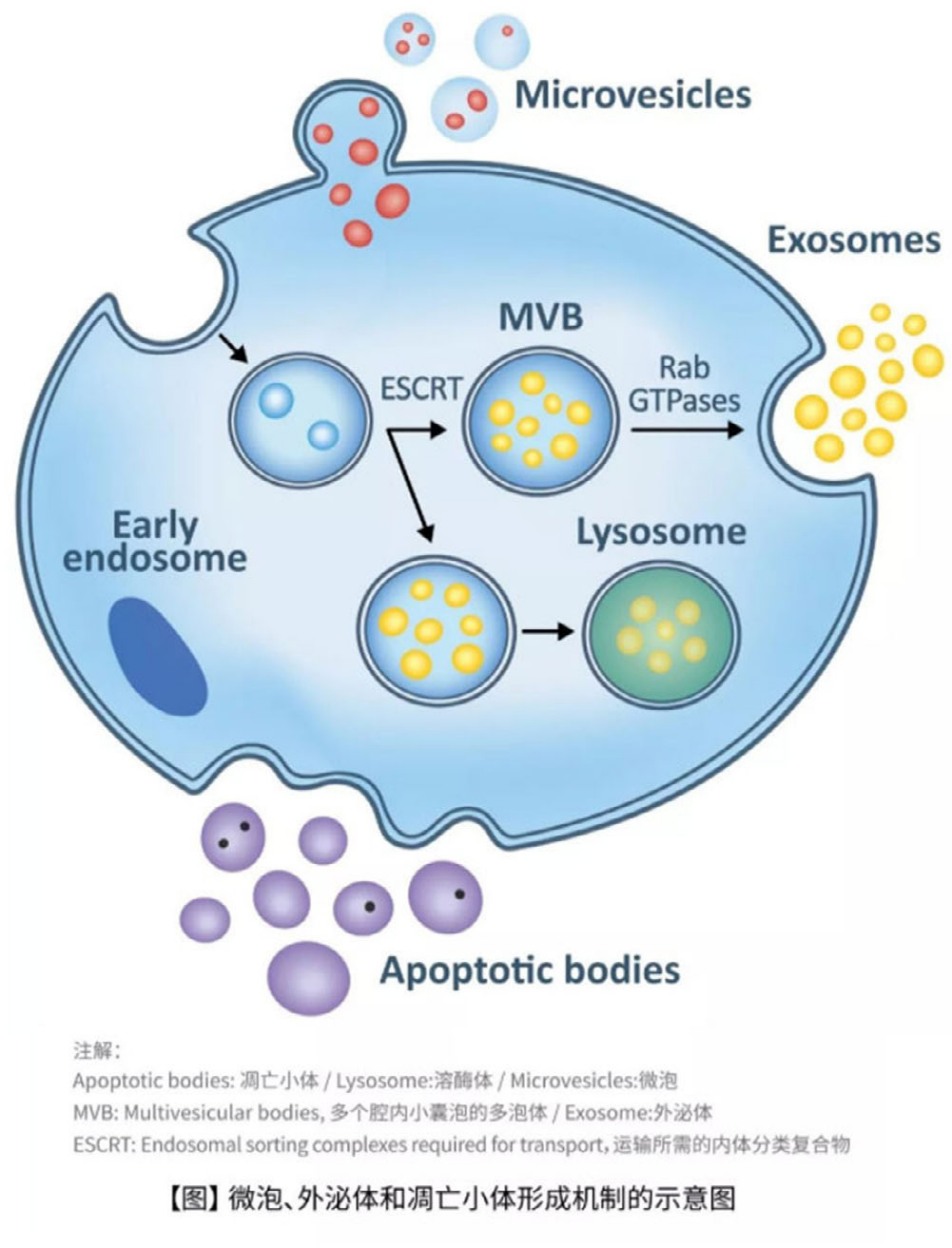
Biology of exosome production and secretion.

Exosomes are born within the endosomal pathway, a network intricately involved in cellular trafficking and sorting. The journey begins with the internalization of extracellular materials into early endosomes.^18^ These early endosomes mature into multivesicular bodies (MVBs) through a complex process guided by endosomal maturation proteins.^19^ Within MVBs, the endosomal membrane undergoes invagination, leading to the formation of intraluminal vesicles (ILVs). This encapsulation of cytoplasmic components within ILVs is a hallmark of exosome biogenesis.^20^ MVBs can either fuse with lysosomes for degradation or, more notably, fuse with the cell membrane for exosome release into the extracellular space (Figure 1). The molecular mechanisms regulating the trafficking of MVBs and the fusion events are tightly controlled. This controlled release allows exosomes to function as carriers of bioactive cargo, facilitating communication between cells and influencing the microenvironment.^21^

The selective loading of cargo into ILVs is a finely tuned process critical for the functional diversity of exosomes.^22^ The Endosomal Sorting Complex Required for Transport (ESCRT) machinery, comprising ESCRT‐0, ESCRT‐I, ESCRT‐II and ESCRT‐III complexes, orchestrates this sorting process. ESCRT‐0 recognizes ubiquitinated cargo, ESCRT‐I and ESCRT‐II facilitate membrane deformation and ESCRT‐III drives membrane scission, leading to ILV formation.^23^, ^24^ Additionally, ESCRT‐independent pathways involving tetraspanins, lipids and heat shock proteins contribute to cargo sorting diversity,^24^ providing alternative avenues for exosome formation.

## 3 STRATEGIES FOR EXOSOME ENGINEERING

### 3.1 Engineering strategies for targeted delivery

**FIGURE 2:**
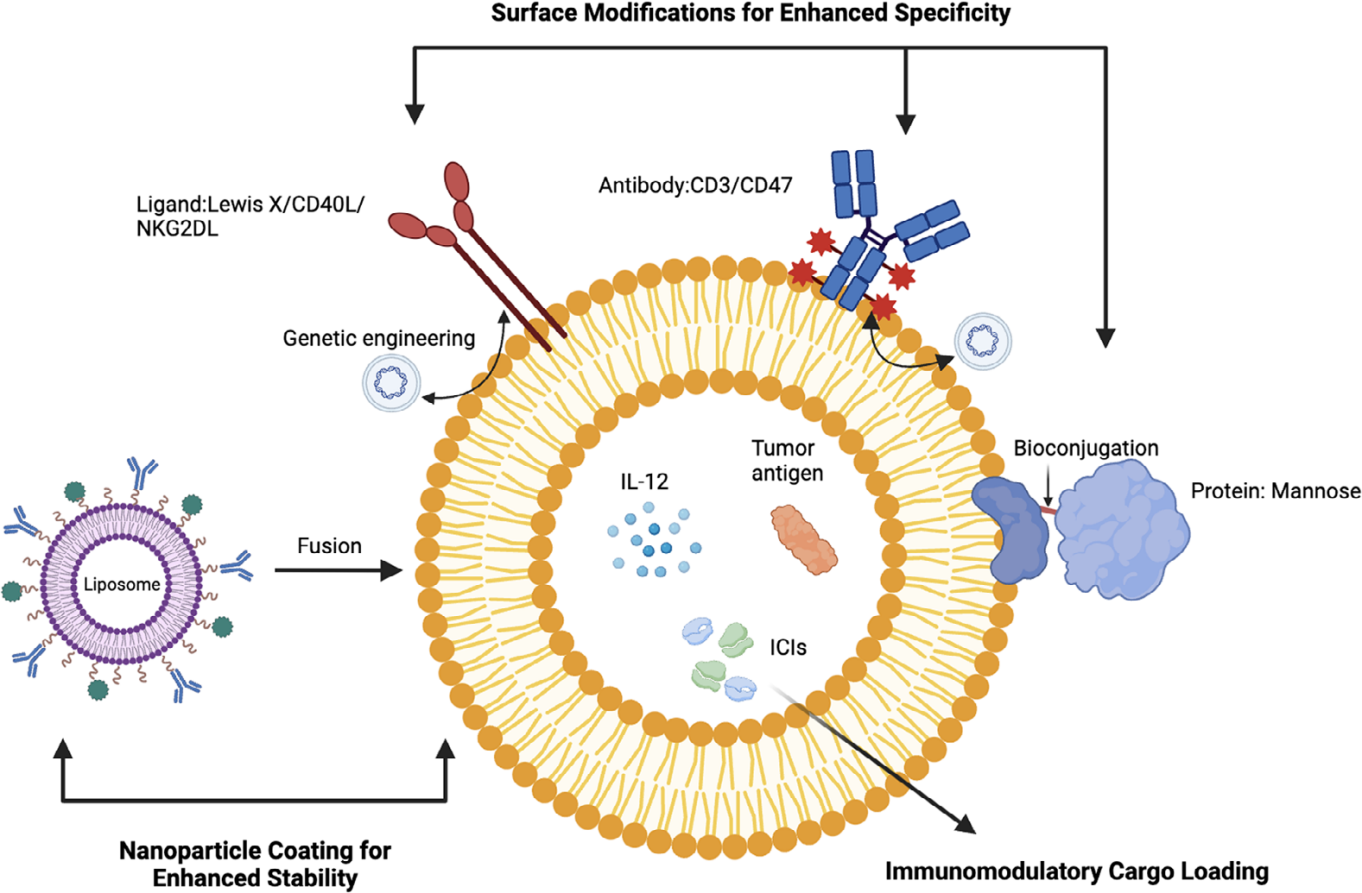
Strategies for exosome engineering. Surface modifications for enhanced specificity, nanoparticle coating for enhanced stability and immunomodulatory cargo loading.

Precision in targeting is essential for effective immunomodulation. Here, the focus shifts to the various engineering strategies employed to enhance the targeted delivery of exosomes to BC cells and immune cells within the tumour microenvironment (TME). This section elucidates specific engineering strategies, each contributing to the intricate orchestration of targeted delivery (Figure 2).

#### 3.1.1 Surface modifications for enhanced specificity

Surface modifications represent a cornerstone in targeted delivery. This involves the strategic incorporation of ligands or antibodies onto the exosome surface, enabling recognition and binding to specific receptors overexpressed on target cells. The section details various surface modification techniques, including bioconjugation and genetic engineering, providing insights into the methodologies employed to equip exosomes with homing capabilities necessary for precise targeting in BC therapy.

Bioconjugation technology is a versatile and indispensable tool in the field of biotechnology and molecular biology. It involves the covalent linkage of two or more different biological molecules to create a functional and often multifunctional entity. The primary aim of bioconjugation is to combine the unique properties of distinct biomolecules, such as proteins, peptides, nucleic acids, or small molecules, to generate innovative and tailored structures with diverse applications.^25^ In recent years, the bioconjugation of small and large molecules with exosome surfaces has become a very interesting and emerging approach for achieving specific goals, therapeutic purposes, in vivo imaging, etc.^26^

It is noteworthy that, in the realm of enhancing the targeting specificity of exosomes toward immune cells, Choi et al. discovered that mannose‐conjugated modified exosomes exhibited a heightened affinity for mannose receptors on dendritic cells (DCs). This phenomenon resulted in an increased uptake of engineered exosomes by DCs.^27^ This discovery opens new possibilities for the application of exosomes in immunotherapy, particularly in augmenting treatment efficacy and minimizing side effects. The incorporation of bioengineered components, such as peptides or proteins, onto exosome surfaces offers notable advantages in BC therapy, enhancing both targeting precision and tissue penetration, thereby promoting increased accumulation within cancerous tissues. However, this strategy is not without challenges. Concerns arise over the potential immunogenicity of bioengineered elements, necessitating careful consideration. Moreover, the complexity associated with the synthesis and characterization of these components poses obstacles that must be addressed. Thorough examination of long‐term effects and safety considerations is imperative to advance the development and application of bioengineered exosomes in BC treatment.^28^

As for genetic engineering, Zhang et al. utilized the CRISPRi system to externally engineer peptides that specifically target tumour‐associated macrophages tumor‐associated macrophages (TAMs) onto the exosome membrane. This modification enabled exosomes to selectively home in on tumour tissues and precisely target M2‐like TAMs, leading to the induction of TAMs polarizing towards the M1 phenotype.^29^ Moreover, the study also highlights the potential of genetically engineered exosomes in targeting DCs. Briefly, exosome producer cells undergo genetic engineering to co‐express a glycosylation domain (GD) inserted into the extensive extracellular loop of CD63, a well‐established scaffold protein for exosomes, along with fucosyltransferase IX (FUT9). This modification results in the production of exosomes that carry Lewis X ligands. The inclusion of Lewis X allows these exosomes to selectively interact with DC‐specific intercellular adhesion molecule‐3 grabbing non‐integrin expressed on the surface of DCs, thereby conferring a heightened specificity for activated DCs.^30^ In addition, exosomes modified with CD40 ligand (CD40L) can specifically target DCs expressing CD40 on their surface, enhancing DC targeting through CD40L‐CD40 interactions.^31^ Similarly, the optimization of exosomes through the incorporation of ULBPs and MICA ligands represents a strategic approach to augment their recognition by natural killer (NK) cells.^32^ It is crucial to comprehend that the activation of the NK receptor group 2 member D (NKG2D) by NKG2D ligands (NKG2DLs) expressed on NK cells induces pivotal immune effector functions, including cytokine production and cellular cytotoxicity.^33^ Notably, NKG2DLs are categorized as ‘stress‐inducible’ molecules and encompass families such as ULBPs and MICA.^34^ In conclusion, these studies present promising avenues for advancing tumour immunotherapy through precision targeting of innate immune cells. The potential to modulate TAMs, DCs and NK cells underscores the versatility and promising applications of genetic engineering in the evolving landscape of cancer treatment. In addition to the aforementioned innate immune cells, the study revealed that exosomes engineered with genetic inserts of programmed death ligand 1/cytotoxic T‐lymphocyte–associated antigen 4 (PD‐L1/CTLA‐4) on their surface could selectively bind to PD‐1 and CD80 receptors on the surface of T cells.^35^ Although this modification leads to immune suppression of T lymphocytes, thus promoting immune escape in tumours and limiting the potential for immunotherapy in cancer. However, it also provides us with a way to enhance the specificity of T cell targeting. This is achieved by genetically engineering exosomes to surface‐insert specific peptides targeting T cells. It is well established that CD28 on T cells binds to CD80/CD86 ligands present on antigen‐presenting cells. This interaction serves to activate and initiate downstream signaling, thereby facilitating T cell function, proliferation and survival.^36^ Therefore, future studies may explore the application of genetic engineering methods to incorporate CD80/CD86 onto the surface of exosomes. This approach aims to construct engineered exosomes that can evade the immunosuppression caused by immune checkpoint molecules such as PD‐L1/CTLA‐4. Moreover, it may enhance the targeting specificity of exosomes against T cells. The significance of these findings lies in the potential to shape the TME, promoting an immune response that is conducive to tumour elimination. Genetic modification of exosome‐producing cells emerges as a promising strategy, offering distinct advantages in tailoring exosomes for enhanced therapeutic efficacy. This approach allows for the expression of specific therapeutic proteins or RNA, enabling the production of customized exosomes with controlled cargo content and release kinetics. However, several challenges accompany this method. Safety concerns associated with genetic modifications necessitate comprehensive investigation into potential long‐term effects. Additionally, challenges related to scalability and regulatory approval for genetically modified exosomes must be addressed to ensure their successful translation into clinical applications.^37^

#### 3.1.2 Nanoparticle coating for enhanced stability

Nanoparticle coating stands as a pivotal strategy within the realm of engineering exosomes, offering a multifaceted approach to enhance their stability, bioavailability and overall performance. This technique involves enveloping exosomes with biocompatible nanoparticles, such as liposomes or polymers, contributing to the robustness of these nanoscale carriers.^38^

The presence of nanoparticle coatings on exosome surfaces imparts distinct properties to these nanocarriers. It can alter the surface charge, influence interactions with biological entities and modulate the release kinetics of cargo molecules. For example, the fusion of tumour‐derived exosomes and cationic liposomes can yield hybrid lipid nanovesicles characterized by positive surface charge.^39^ Given that cationic liposomes tend to bind more effectively to target cell membranes with a negative surface charge, this leads to enhanced absorption of nanoparticle‐coating exosomes by the target cells.^39^ Fascinatingly, diverse liposomes exhibit distinct properties, resulting in variations in the characteristics of engineered exosomes. In a study by Han et al., fusion nanocarriers composed of photothermal sensitive liposomes and exosomes were employed. This design enabled engineered exosomes to achieve tumour targeting through the upregulation of vascular cell adhesion molecule‐1 expression in tumour tissues, triggered by photothermal effects.^40^ Interestingly, Cheng et al. devised hybrid therapeutic nanovesicles through the fusion of genetically engineered exosomes with thermosensitive liposomes loaded with drugs.^41^ In essence, genetic engineering techniques were employed to induce the overexpression of CD47 on the exosome surface, which were then fused with thermosensitive liposomes. It is noteworthy that the elevated CD47 levels on various tumour cells activate a “don't eat me” signal, shielding the exosomes from phagocytosis by the mononuclear macrophage system.^42^ As a result, the hybrid therapeutic nanovesicles demonstrated prolonged blood circulation and achieved preferential accumulation at the tumour sites simultaneously. These results suggest that multiple engineering techniques can be combined to improve targeted exosome delivery. The hybrid approach of combining exosomes with synthetic nanoparticles offers a synergistic blend of advantages. This strategy capitalizes on the strengths of both systems, improving overall stability, cargo loading efficiency and targeting capabilities for enhanced therapeutic outcomes. However, challenges exist in the form of complex fabrication processes and concerns regarding the potential toxicity of synthetic nanoparticles. For example, the treatment method of “Dacarbazine‐primed carbon quantum dots coated with BC cell‐derived exosomes” represents an innovative approach that enhances the treatment of BC. This strategy involves coating dacarbazine‐primed carbon quantum dots onto the surface of exosomes derived from BC cells. The utilization of dacarbazine pre‐treatment enhances therapeutic efficacy, while the source of exosomes imparts specificity to the treatment.^43^ However, it is essential to note potential drawbacks, such as the possible toxicity associated with synthesized carbon quantum dots and the complexity of the preparation process, which may pose challenges for clinical applications.^44^ Therefore, a comprehensive evaluation of safety, efficacy, and the resolution of potential technical challenges are crucial for advancing the development of this therapeutic strategy.

In summary, nanoparticle coating emerges as a powerful tool in engineering exosomes, enhancing their stability and expanding their potential applications in drug delivery, diagnostics and theranostics. While nanoparticle coating significantly enhances stability, challenges remain, including potential alterations to exosome functionality and the need for fine‐tuning coating materials. Future research directions should focus on optimizing coating techniques, ensuring minimal interference with exosome properties and addressing concerns related to potential immunogenicity or toxicity associated with the nanoparticles used.

### 3.2 Immunomodulatory cargo loading

This section unravels the diverse cargo options for loading into engineered exosomes, including cytokines, immune checkpoint inhibitors (ICIs) and tumour antigens. Molecular insights into cargo loading strategies are discussed, emphasizing their implications in reprogramming the immune microenvironment in BC. The review sheds light on the strategic choices in cargo loading to achieve optimal therapeutic outcomes (Figure 2).

Within the realm of cytokines, interleukin‐12 (IL‐12) is acknowledged as a pro‐inflammatory cytokine and has been extensively explored as a promising candidate for cancer immunotherapy.^45^ Notably, engineered exosomes over‐loaded with IL‐12 exhibited activation of DCs, emerging as effective adjuvants to promote the induction of specific anti‐tumour responses in immunotherapy regimens.^46^ In another study, a stimulator of interferon genes (STING) agonist, capable of activating the STING pathway to induce type I interferon responses and cytokine production, was loaded into exosomes. These engineered exosomes, upon uptake by DCs, activated CD8+ T cell‐mediated anti‐tumour immune responses. This highlights the potential of engineered exosomes as carriers for STING agonists in promoting anti‐tumour immune therapy.^47^ In terms of future directions, the potential for further clinical and foundational research is highlighted. The application of engineered exosomes in cancer immunotherapy opens avenues for refining treatment strategies and exploring novel combinations of cytokines and immune modulators. Additionally, investigations into the specific mechanisms underlying the observed immune responses could deepen our understanding and inform the design of more targeted and effective therapeutic interventions.

For ICIs, PD‐1 antibodies were loaded onto exosome carriers using the streptavidin‐biotin method, enabling the engineered exosomes to release suppressed T lymphocytes.^48^ Additionally, in the recent study by Chen et al., the authors detail the development and therapeutic application of exosomes overexpressing a high‐affinity variant of human PD‐1 (havPD‐1) as ICIs and drug nanocarriers for tumour immunotherapy and chemotherapy. This achievement necessitated the establishment of a tumour cell line lacking human leukocyte antigen class I and PD‐L1, while simultaneously overexpressing the high‐affinity havPD‐1.^49^ While the current focus primarily centers on PD‐1/PD‐L1 inhibitors, there is a growing need to explore and successfully load various ICIs onto exosomes. This expansion is crucial for achieving a more comprehensive and precise immune therapeutic approach. In the context of diseases like BC, known for intricate immune escape mechanisms, such advancements hold considerable promise. Future research directions should encompass the identification and investigation of immune checkpoints applicable to BC, broadening the scope of immune therapeutic interventions. Optimization of loaded immune checkpoints on exosomes is imperative for enhancing treatment efficacy and specificity. Additionally, a deeper understanding of the role and mechanisms of exosomes in immune modulation is essential, unveiling further therapeutic potential and advantages.

The utilization of exosomes derived from tumour cells for the development of tumour vaccines is a promising avenue in cancer immunotherapy. The presence of numerous tumour antigens within these exosomes provides a robust foundation for the stimulation of potent anti‐tumour immunity, as highlighted by the works of various researchers.^50^, ^51^ One such tumour antigen, fibroblast activating protein‐α (FAP), has garnered attention due to its overexpression in cancer‐associated fibroblasts (CAFs) across more than 90% of human tumour tissues.^52^ In a notable study by Hu et al., a novel approach was introduced wherein FAP gene‐engineered tumour cell‐derived exosome‐like nanovesicles (eNVs‐FAP) were developed as a convenient and scalable tumour vaccine.^53^ The significance of this study lies in the mechanistic insights provided by the analysis of eNVs‐FAP. It was revealed that these nanovesicles played a crucial role in promoting the maturation of DCs. Additionally, they were found to enhance the infiltration of effector T cells into target tumour cells and FAP‐positive CAFs (FAP^+^CAFs). Moreover, eNVs‐FAP demonstrated the potential to reduce the proportion of immunosuppressive cells within the TME.^53^ While this research showcases a notable advancement in the field of cancer immunotherapy, several areas merit further exploration for future studies. Firstly, a deeper understanding of the specific mechanisms underlying the interactions between eNVs‐FAP and immune cells is essential. Unraveling the intricate details of how these nanovesicles modulate DC maturation and T cell infiltration could pave the way for targeted optimization. Additionally, exploring the versatility of exosomes from various tumour types and their antigenic contents could uncover a broader spectrum of tumour vaccines. Comparative studies between different tumour‐derived exosomes may elucidate unique features and antigens, providing a basis for developing personalized and highly effective immunotherapeutic strategies.

Loading exosomes with therapeutic agents, such as chemotherapeutic drugs or small interfering RNA (siRNA), represents a promising strategy to bolster their anti‐cancer properties through targeted drug delivery to cancer cells. This approach holds distinct advantages in precision medicine. However, challenges arise in terms of loading efficiency, with variability in the stability of loaded cargo within exosomes. The loading process itself introduces complexities, altering the natural properties of exosomes and potentially impacting their biodistribution and cellular uptake. Striking a balance between efficient cargo loading and preserving the inherent characteristics of exosomes is essential for realizing the full therapeutic potential of this strategy.^54^

## 4 ENGINEERED EXOSOMES FOR IMMUNOMODULATION IN BC

**FIGURE 3:**
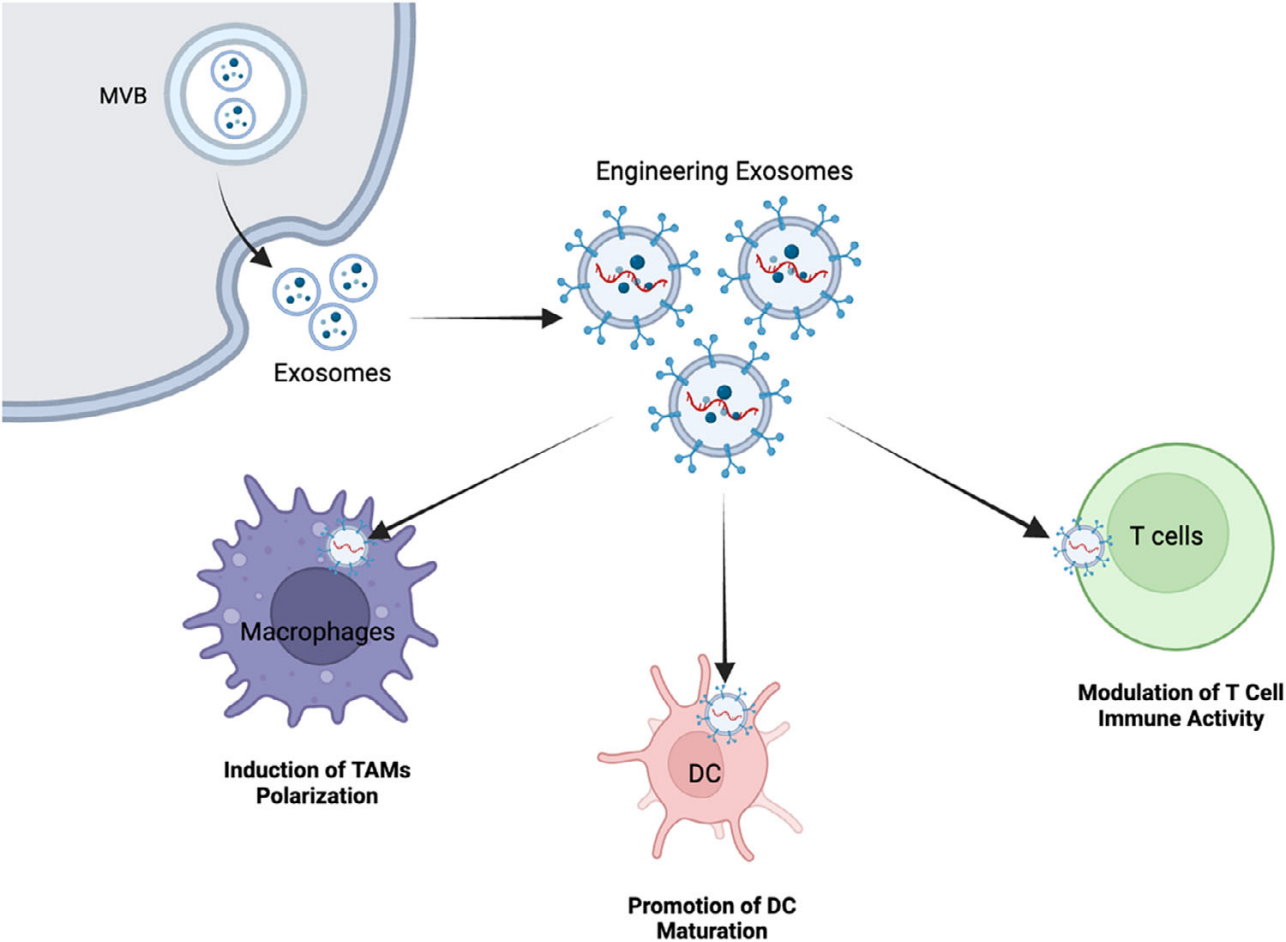
**Engineered exosomes for immunomodulation in breast cancer**. Engineered exosomes actively contribute to inducing tumour‐associated macrophage (TAM) polarization, enhancing dendritic cell (DC maturation and modulating T‐cell immune activity.

This chapter will analyze the immunomodulatory role of engineered exosomes in BC, including inducing TAM polarization, promoting DC maturation and regulating T cell immune activity (Table 1, Figure 3).

**TABLE 1:** Engineered exosomes for immunomodulation in breast cancer.

Engineering strategy	Cargoes or Surface protein	Target cell	Mechanism	Ref.
Cargo loading	miR‐511‐3p	TAM	Inhibiting tumour growth by carrying −511‐3p to reprogram TAM into M1‐like macrophages	^62^
Surface modification	IL4RPep‐1	TAM	Improving the targeting specificity of TAMs	^62^
Surface modification	aCD47 and aSIRPα	TAM	Enhancing the phagocytosis capacity of macrophages towards breast cancer cells	^64^
Cargo loading	PH20	TAM	Enhancing M1 polarization in TAMs	^68^
Cargo loading	ICD inducers, ELANE and Hiltonol	DC	Promoting in situ activation of cDC1s	^74^
Surface modification	CD40L	DC	Enhancing the targeting of exosomes to DCs	^31^
Surface modification	CD62L	T Cell	Enhancing the targeting of exosomes to TDLN	^80^
Surface modification	OX40L	T Cell	Facilitating the penetration of CD8+ T cells and alleviating the immune suppression by Tregs	^80^
Surface modification	anti‐human CD3 and anti‐human HER2 antibodies	T Cell	Enabling dual targeting of T cells for CD3 and HER2‐positive breast cancer	^84^

### 4.1 Induction of TAM polarization by engineered exosomes

TAMs represent a subset of immune cells within the intricate landscape of the TME, actively participating in pivotal processes such as tumourigenesis, development and metastasis.^55^, ^56^, ^57^ The two distinctive subtypes of TAMs, namely the classically activated M1 type and the selectively activated M2 type, play divergent roles in influencing the tumour milieu.^58^ M1‐type macrophages demonstrate a proclivity for secreting inflammatory cytokines, thereby exerting anti‐tumour effects. In contrast, M2‐type macrophages, characterized by their secretion of anti‐tumour cytokines, paradoxically contribute to tumour progression.^59^ Notably, within the TME, TAMs often exhibit a propensity for polarization towards the M2 type.^59^ This underscores the significance of regulating TAM polarization as a strategic approach to impede the progression of BC. In essence, understanding and modulating the delicate balance between M1 and M2 polarization within TAMs emerge as critical considerations in devising interventions aimed at slowing the development of BC.

Prior investigations have established that miRNAs play a pivotal role in modulating the polarization of TAMs.^60^ This revelation opens up a new avenue for leveraging miRNAs as a novel approach to tumour immunotherapy. Furthermore, exosomes, as emerging carriers of miRNAs, offer a promising means to actively engage in the immune regulation of tumours. The integration of miRNAs into the realm of immunotherapy represents a paradigm shift, and exosomes serve as innovative vehicles for the targeted delivery of these regulatory molecules.^61^ Numerous studies have explored the use of engineered exosomes loaded with miRNAs to finely tune the polarization of TAMs, thereby exerting a direct impact on the intricate processes governing the development of BC. For example, Gunassekaran et al. conducted a groundbreaking study wherein they transfected exosomes derived from M1 macrophages with miR‐511‐3p. Additionally, they engineered these exosomes with a modification of IL4RPep‐1 (an IL4 receptor‐binding peptide) to target TAMs expressing the IL‐4 receptor, resulting in the creation of IL4R‐Exo (si/mi).^62^ Notably, the IL‐4R holds significance in the context of TAMs, as its elevated expression on their surface facilitates the binding with IL‐4, thereby promoting TAM polarization towards the M2 phenotype and consequently fostering tumour development.^63^ By virtue of its targeted design, IL4R‐Exo (si/mi) can improve the targeting specificity of TAMs. At the same time, IL4R‐Exo (si/mi) inhibits tumour growth by carrying miR‐511‐3p to reprogram TAM into M1‐like macrophages.^62^ The above research findings highlight the innovative application of genetically engineered exosomes in regulating BC by enhancing their targeting capabilities towards TAMs through surface modification. Furthermore, the study underscores the critical role played by tailored loading of immunomodulatory cargo into exosomes, contributing to the overall effectiveness of these engineered exosomes in cancer immunotherapy. The findings of this study pave the way for further exploration and refinement of targeted exosome‐based therapies in the realm of cancer immunotherapy.

Regarding surface modification for enhanced specificity, in the regulation of TAMs in BC, bioconjugation techniques have found application alongside genetic engineering technologies. Exosomes modified with azide, originating from M1 macrophages, are synthesized into exosome nano‐bioconjugates by conjugating them with antibodies specific to CD47 and SIRPα (aCD47 and aSIRPα), which are modified with dibenzocyclooctyne, using pH‐sensitive linkers.^64^ As a result, within the acidic TME, the benzoic‐imine bonds of the nano‐bioconjugates undergo cleavage, liberating aSIRPα and aCD47. These components individually inhibit SIRPα on macrophages and CD47, leading to the elimination of the “don't eat me” signalling. Consequently, this process enhances the phagocytosis capacity of macrophages towards BC cells.^64^ For future research directions, a deeper exploration into the optimal design of these bioconjugated exosomes, considering factors such as linker stability and specificity, could provide insights into refining the approach. Additionally, investigate the broader applicability of this strategy in various cancer types and the potential impact on the broader immune.

In the realm of loading immunomodulatory cargo, hyaluronic acid, alongside miRNAs, can be integrated into exosomes, exerting an influence on the polarization of TAMs. It's noteworthy that high molecular weight hyaluronic acid has demonstrated the ability to inhibit M1 polarization while promoting M2 polarization.^65^, ^66^ To counteract this effect, hyaluronidase comes into play by degrading high molecular weight hyaluronic acid into its low molecular weight counterpart, thereby mitigating the M2‐induced immunosuppressive response.^67^ In a groundbreaking approach, Feng et al. pioneered an advanced exosome‐based drug delivery system termed Exos‐PH20‐FA. Utilizing genetic engineering, they expressed human hyaluronidase (PH20) and employed self‐assembly techniques to integrate folic acid (FA) into the exosomes. Within the context of BC, Exos‐PH20‐FA efficiently transformed polymer hyaluronic acid into a low molecular weight form. This transformation significantly enhanced M1 polarization in TAMs, ultimately contributing to an amplified anti‐tumour effect.^68^ In the latest research, Li et al. reported a genetically engineered artificial exosome‐constructed hydrogel. Initially, artificial exosomes were prepared through an extrusion method from genetically engineered M1‐type macrophages with overexpressed Siglec‐10, denoted as SM1Aexo. The as‐prepared SM1Aexo were further chemically modified through a Schiff base reaction, wherein they underwent chemical modification with sodium alginate oxide (OSA). The reaction involved the aldehyde groups of OSA and the amine groups of macrophage membrane proteins, resulting in the formation of a hydrogel, referred to as O‐SM1Aexo@M hydrogel. The O‐SM1Aexo@M hydrogel can target peritoneal macrophages and polarize M2‐type macrophages into the M1 phenotype, exerting anticancer effects in triple‐negative BC.^69^ Similarly, Yu et al. have engineered exosomes derived from M1‐type macrophages, referred to as OX40L (tumour necrosis factor receptor superfamily member 4) M1‐exos. These exosomes activate the adaptive immune response through the OX40/OX40L pathway. Furthermore, they can reprogram M2‐like TAMs into M1‐like macrophages, achieving an effective synergistic effect between innate and adaptive immunity. This approach has demonstrated a potent therapeutic effect in a mouse BC model.^70^

In summary, this research signifies a critical step toward understanding and manipulating the immune response in BC through exosome‐mediated immunomodulation. Future research directions may explore optimizing cargo selection, refining engineering techniques, and investigating the broader implications of exosome‐based therapies for personalized BC treatment.

### 4.2 Promotion of DC maturation by engineered exosomes

DCs play a pivotal role in initiating and regulating both innate and adaptive immunity within the TME.^71^ Consequently, various DC‐targeted vaccines have been designed and are undergoing evaluation in numerous clinical trials to enhance cancer immunotherapy.^72^ The targeted delivery of antigens and adjuvants to DCs in vivo is a crucial strategy in the development of DC vaccines.^73^

Building on this, Huang et al. engineered an in situ DC vaccine, termed HELA‐Exos, by loading immunogenic cell death immunogenic cell death (ICD) inducers, human neutrophil elastase (ELANE) and Hiltonol (TLR3 agonist) into BC‐derived exosomes engineered with α‐lactalbumin (α‐LA). HELA‐Exos demonstrated robust antitumour efficacy in both mouse models and human BC organoids. This effect was attributed to the promotion of in situ activation of conventional type 1 DCs (cDC1s), leading to enhanced tumour‐reactive CD8+ T cell responses.^74^ The strategy proposed here is promising for generating an in situ DC‐primed vaccine and can be extended to various types of cancers. The study highlights the significance of engineering exosomal cargoes to regulate DC maturation, emphasizing the importance of this approach in BC. Future research should aim to identify additional immunomodulatory cargoes capable of modulating the activity of DCs in BC. This involves exploring different antigens and molecular signals to stimulate DC maturation, as well as optimizing exosome engineering for enhanced efficacy in modulating immune responses. Additionally, exosomes modified with CD40L can enhance the targeting of exosomes to DCs.^31^ This engineered exosome, through CD40L modification, may have the potential to boost the immune response against BC. Further exploration is warranted to investigate the effects of these engineered exosomes and their ability to enhance immune responses in BC. Moreover, the combination of CD40L modification with the engineering of exosomal cargoes holds the potential to synergistically stimulate DC maturation, thereby increasing their ability to elicit cytotoxic effects against BC.

### 4.3 Modulation of T cell immune activity by engineered exosomes

T lymphocytes constitute a crucial component of the immune system with profound implications for tumour dynamics. Within the TME, T cells can be broadly categorized based on surface molecules into three groups: CD4+ T cells, CD8+ T cells and regulatory T cells (Tregs).^75^ Among these, CD4+ T cells and CD8+ T cells predominantly exert anti‐tumour effects in BC. Conversely, Tregs function as immunosuppressive cells, dampening the body's anti‐tumour immune response and thereby fostering the progression of BC.^76^

Enhancing the specific targeting of exosomes to T lymphocytes has been a challenging task. Interestingly, recent advances in BC research have shed light on this issue. Unlike other cancers, BC can lead to tumour‐draining lymph node (TDLN) metastasis, which is the most common type of metastasis.^77^, ^78^ Moreover, TDLNs serve as primary immune organs for cancer immunity, where tumour antigens and leukocytes are initially drained.^79^ Therefore, improving the targeting of exosomes to TDLNs could enhance the immunomodulation of T lymphocytes within them. To achieve this, current research has specialized in designing exosomes targeted for lymph nodes. Through genetic engineering, these exosomes are modified to express CD62L on their surface, guiding white blood cells to migrate and home to lymph nodes.^80^ Additionally, the combination of targeted delivery engineering strategies with exosomal cargo engineering has been a crucial approach to enhancing the immunomodulatory potential of exosomes. Therefore, while surface‐modifying with CD62L, molecules such as OX40L, involved in T cell expansion and regulation of T cell suppression,^81^, ^82^ are also engineered onto exosomes. This engineered exosome activation of effector T cells and inhibition of Treg induction amplifies the anti‐tumour immune response, suppressing the development of BC. In addition to targeting lymph nodes for specific T cell modulation, more direct approaches have been developed, such as surface modification with CD3 antibodies to target T lymphocytes.^83^ Although this research is conducted in BC, these engineered exosomes can also target T cells in other types of cancer. Consistent with the studies mentioned earlier, efforts have been made to enhance the specificity of exosomes. OX40L has been added to facilitate the penetration of CD8+ T cells and alleviate the immune suppression by Tregs. Notably, this engineered exosome is equipped with a monoclonal antibody targeting the epidermal growth factor receptor. This feature enables the exosomes to selectively target tumour tissues, thereby boosting the immune response against cancer.^83^ In addition, another engineered exosome has been designed through genetic manipulation to display anti‐human CD3 and anti‐human HER2 antibodies, enabling dual targeting of T cells for CD3 and HER2‐positive BC.^84^ This work demonstrates the preclinical feasibility of utilizing endogenous exosomes for targeted BC immunotherapy. In addition, surface modification of IL‐2 on DC‐derived exosomes enables targeted delivery of engineered exosomes to lymphocytes and enhances binding to IL‐2 receptors on CD8+T cells, thereby inducing CD8+T cell activation.^85^

Looking ahead, future clinical and basic research in BC could focus on translating these engineered exosome strategies into clinical settings. Rigorous evaluation in clinical trials, exploring safety and efficacy across diverse patient populations, will be essential. Additionally, deeper investigations into the mechanisms underlying exosome‐T cell interactions and their impact on broader immune responses could provide crucial insights for refining these approaches.

## 5 PROSPECTIVE APPLICATIONS OF ENGINEERED EXOSOMES IN BC IMMUNOTHERAPY

Immunotherapy stands out as one of the most promising approaches for treating BC. However, past research has indicated that BC is often considered an “immune cold” tumour.^86^ This designation stems from the lower presence of lymphocytes infiltrating the immune microenvironment in secondary BC, rendering it less responsive to immunotherapeutic interventions.^87^ Hence, there is a critical need to enhance immunotherapy methods to improve their efficacy in BC. In light of the distinctive attributes of engineered exosomes outlined earlier, these tailored exosomes have emerged as a crucial tool in adjuvant immunotherapy for BC. Their unique properties position them as valuable assets in augmenting the effectiveness of immunotherapeutic interventions against BC.

### 5.1 Engineered exosomes as drug delivery vehicles

For effective cancer therapy, it is crucial to have targeted drug delivery vehicles with minimal immunogenicity and toxicity. In this study, a strategy was employed to mitigate immunogenicity and toxicity by utilizing mouse immature DCs (imDCs) for exosome production. Through the genetic modification of imDCs, a fusion peptide was created by integrating the exosome membrane protein (Lamp2b) with the αv integrin‐specific iRGD peptide, enhancing tumour‐targeting capabilities. This engineered exosome was then loaded with Doxorubicin (Dox) using electroporation, achieving an impressive encapsulation efficiency of up to 20%. Notably, iRGD exosomes exhibited highly efficient targeting and Dox delivery specifically to alpha v integrin‐positive BC cells in vitro. This innovative approach holds promise for advancing targeted and low‐toxicity drug delivery systems in cancer therapeutics.^88^ While the focus of this study is on loading chemotherapeutic agents into exosomes, it opens up a new avenue for immunotherapy agents. Interestingly, a recent study has explored the utilization of exosomes for delivering immune activators. Specifically, the RIG‐I pathway can be triggered by RNA containing 5′ triphosphate, resulting in the release of type I interferon and immune activation. Therefore, RIG‐I agonists have been investigated as a means to elicit immune responses against cancer, showcasing the potential of exosomes in the field of immunotherapy. This demonstrates the versatility of exosome‐based platforms in delivering various therapeutic agents for different treatment modalities.^89^, ^90^ Additionally, Peng et al. successfully loaded RIG‐I agonists into exosomes derived from red blood cells. The engineered exosomes demonstrated the capability to selectively target aggregation within BC cells. Upon reaching the target cells, they activated the RIG‐I pathway, ultimately leading to cell death in both mouse and human BC cells. This innovative approach highlights the potential of utilizing EVs for targeted delivery of immune activators, providing a promising strategy for cancer treatment.^91^ This innovative approach not only emphasizes the versatility of exosome‐based platforms but also suggests a promising strategy for targeted delivery of immune activators, further expanding the therapeutic possibilities in cancer treatment. Overall, these studies collectively contribute to advancing the field of cancer therapy by harnessing the potential of engineered exosomes for targeted drug delivery and immunotherapy. Future research directions could explore optimizing these strategies for clinical applications and addressing challenges associated with large‐scale production and clinical translation.

### 5.2 Engineered exosomes as vaccines for BC immunotherapy

Tumour vaccines leverage elevated concentrations of tumour antigens to activate DCs. Activated DCs, in turn, prompt T cells to generate an anti‐tumour immune response, effectively suppressing tumours.^92^ Exosomes derived from tumours play a pivotal role by carrying tumour antigens and presenting them to antigen‐presenting cells, thereby instigating an antitumour immune response.^8^, ^93^ To enhance their capacity to induce immune responses, tumour‐derived exosomes can undergo surface modification or cargo loading.^13^, ^94^ Recently, numerous studies have utilized tumour‐derived engineered exosomes as a foundation for immunological vaccines targeting BC. For example, the in situ DCs‐primed vaccine (HELA‐Exos), loaded with ICD inducers, ELANE and Hiltonol, has been confirmed in in vivo studies to enhance the immune response against BC by stimulating cDC1s, thereby increasing CD8+ T cell‐mediated immunity.^74^ These research findings underscore the significance of engineered exosomes in the development of DC‐targeted vaccines. In addition to DC‐targeted vaccines, more research is exploring the application of T cells‐targeted vaccines in BC. Muzaffer et al. pioneered the development of exosome‐based vaccines by incorporating two immunoadjuvant agents, a TLR9 ligand (K‐type CpG ODN) and a TLR3 ligand (p(I:C)), into exosomes derived from BC cells. Engineered exosomes co‐encapsulating both ligands exhibited enhanced immunostimulatory properties, activating antigen‐specific primary and memory T cell responses and effectively suppressing BC growth in vivo. Collectively, these findings suggest that an exosome‐based therapeutic vaccine can stimulate robust cellular and humoral anti‐tumour immunity, demonstrating effectiveness in reversing established tumours.^95^ This approach shows promise as a personalized strategy for tumour therapy with potential clinical applications. Future research directions may involve refining and optimizing the engineering of exosomes for enhanced specificity, exploring combination therapies, and conducting more extensive clinical trials to validate their efficacy and safety in diverse patient populations. Additionally, investigations into the underlying mechanisms of exosome‐mediated immune responses could provide insights for further therapeutic advancements.

While engineered exosome vaccines may activate the immune system, especially T cells, to attack and eliminate tumour cells, this can lead to long‐term anti‐tumour immune effects. Well‐designed vaccines are expected to induce the immune system to develop persistent immune memory against tumours, enabling a more effective response when facing the same tumour in the future.^15^ However, prolonged use of engineered exosome vaccines may result in excessive activation of the immune system, triggering inappropriate immune responses and even potentially leading to the onset of autoimmune diseases. Continued exposure to exosome vaccines may cause the immune system to become tolerant to the signals they generate, thereby weakening immune responses.^96^ To comprehensively understand the long‐term effects of engineered exosome vaccines on the immune system, large‐scale and long‐term clinical studies are necessary. Such research helps evaluate the long‐term safety, effectiveness, and overall impact on the immune status of patients.

### 5.3 Engineered exosomes from CAR‐T cell for BC immunotherapy

CAR‐T cell therapy, a form of immunotherapy created through the genetic engineering of CAR into T cells, has emerged as a novel and precise approach to cancer treatment.^97^, ^98^ Despite its remarkable targeting efficacy, the application of CAR‐T therapy is hampered by associated toxic side effects, including cytokine release syndrome (CRS) and CAR‐T‐related encephalopathy syndrome (CRES).^99^ These side effects, albeit manageable, highlight the need for alternative strategies to enhance safety and efficacy.

As a potential solution to mitigate some of the limitations of CAR‐T therapy, CAR‐T cell‐derived exosomes have emerged as a novel therapeutic modality.^100^ Exosomes released by CAR‐T cells carry CAR on their surface, expressing a high level of cytotoxic molecules that endow them with potent anti‐tumour capabilities.^101^ Notably, CAR exosomes lack PD‐1 expression, offering an advantage over CAR‐T cells, as their anti‐BC immune effect remains unaffected by recombinant PD‐L1 treatment. In a preclinical in vivo model of cytokine release syndrome, the administration of CAR exosomes demonstrates a relatively safe profile when compared to CAR‐T therapy.^101^ This suggests that engineered exosomes expressing CAR obtained through T‐cell modification could represent a promising and safer strategy for BC treatment. The pivotal aspect of this research is centred on the identification of target proteins exclusive to BC. Building on the observation of mesenchymal (MSLN) overexpression in triple‐negative BC (TNBC),^102^ Yang et al. pioneered the development of MSLN‐targeted CAR‐T exosomes for treating TNBC. Significantly, these targeted CAR‐T exosomes demonstrated remarkable efficacy in restraining tumour growth in both endogenous and exogenous MSLN‐positive TNBC models.^103^ Their mechanism of action involved the induction of tumour cell death through the release of perforin and granzyme B. This targeted therapeutic approach showcases the potential of tailored CAR‐T exosomes in addressing specific BC subtypes, exemplifying a targeted and effective strategy for TNBC treatment.

Moving forward, further exploration is warranted in identifying specific target proteins exclusively expressed in BC, ensuring the precision and efficacy of CAR exosomes. Additionally, ongoing research should focus on expanding the application of CAR exosomes to various BC subtypes and investigating their potential in combination with other therapeutic modalities. The ultimate goal is to advance this novel approach from preclinical studies to clinical trials, offering a transformative and personalized strategy for BC immunotherapy.

### 5.4 Combination therapy of engineered exosomes with other immunotherapies

In a groundbreaking study, Tian et al. harnessed the potential of a brain tumour‐targeting cyclic RGDyK peptide to modify the exosome surface. Simultaneously, they ingeniously loaded these exosomes with siRNA targeting PD‐L1, introducing a powerful tool for immune checkpoint blockade. This engineered exosome not only elevates the precision of tumour targeting but also remarkably amplifies the efficacy of ICIs.^104^ In a parallel exploration, a dual‐targeting strategy emerged, wherein the exosome surface underwent simultaneous modification with two antibodies—anti‐PD‐L1 and anti‐CD40. This strategic maneuver sought to simultaneously engage with both tumour cells and DCs. By activating DCs and suppressing PD‐L1 expression on tumour cells, the combined cancer immunotherapy exhibited a synergistic effect, showcasing a notable enhancement in inhibiting tumour growth.^105^ The culmination of these findings underscores the potential of synergistic therapies, fusing engineered exosomes with other immunotherapies, paving the way for advanced cancer treatment.

In BC, Wu et al. investigated the concurrent use of DC‐derived exosomes along with surface‐modified IL‐2 and ICIs. The findings indicated that the combination of engineered exosomes and ICIs rendered tumour cells more responsive to the effects of ICIs. Mechanistically, this combination led to an augmentation in the number of tumour‐infiltrating lymphocytes and activated T cells in the spleen.^85^ This provides a viable strategy for the treatment and prevention of metastatic BC.

## 6 CONCLUSIONS AND FUTURE CHALLENGES

Exosomes, versatile in their distribution and immune characteristics, offer a unique potential in BC immunotherapy by effortlessly navigating tissue barriers. However, exosome‐based BC therapy faces significant challenges, including limited targeting efficiency hindering precise tumour navigation and technical difficulties in cargo loading and controlled release crucial for therapeutic success. Immunogenicity and safety concerns complicate clinical application, demanding thorough investigation and mitigation. The lack of standardized methods for isolating and producing therapeutic exosomes impedes reproducibility, creating a substantial barrier to clinical translation. Biological barriers, exemplified by the blood‐brain barrier, further limit exosome delivery, reducing therapeutic potential.^106^ In response, ongoing research focuses on innovative strategies, including engineered targeting ligands to enhance exosome specificity and advanced cargo loading techniques (e.g. electroporation and sonication) for optimized release. Immunomodulatory approaches, such as surface modification, address immunogenicity concerns. Active pursuit of standardization protocols for the isolation and production of therapeutic exosomes, promoting consistency and reliability in research and clinical applications. This includes the development of innovative strategies such as nanomaterial‐assisted isolation and the utilization of techniques like extracellular vesicle detection to improve the overall process.^107^, ^108^, ^109^ Researchers explore inventive delivery systems to overcome biological barriers, improving exosome distribution. This collective effort seeks to advance the field and unlock the full therapeutic potential of exosomes in BC therapy.

In our exploration of this frontier, we delve into diverse strategies for loading therapeutic agents into exosomes and augmenting their targeting precision. A comprehensive analysis is presented, highlighting the advantages and limitations of each method. Recent experimental studies showcasing the application of engineered exosomes in BC immunotherapy further underscore the dynamic nature of this field, with ongoing research providing evolving insights and promising prospects.

Despite these advancements, the current application of engineered exosomes in BC immunotherapy remains predominantly within the realm of preclinical studies, posing a substantial hurdle in transitioning these findings to clinical practice.^110^ The inherent heterogeneity of exosomes introduces a critical consideration, as excessive modifications may compromise their overall effectiveness.^110^ Overcoming challenges related to achieving optimal loading efficiency and enabling mass production is crucial for the future clinical integration of engineered exosomes.^13^ Each method of exosome processing brings its own set of limitations, emphasizing the necessity for scalable production methods that are pivotal for clinical applications. This underscores the imperative need for continued research into refining engineering and production techniques. Additionally, scaling up exosome production for clinical use presents challenges including low yield, purity concerns, reproducibility issues, adapting to bioreactors, regulatory compliance, storage stability, cost and scalable analytics.^9^ Potential solutions to facilitate the transition from laboratory to clinical settings may involve optimizing culture conditions, adhering to GMP standards, implementing robust purification steps, standardizing protocols, using scalable bioreactors, engaging with regulatory agencies early, developing stability protocols, identifying cost‐effective strategies and implementing scalable analytics like NTA or mass spectrometry. A multidisciplinary approach is crucial for a successful transition to clinical settings.

Moreover, the clinical application of engineered exosome vaccines may encounter regulatory and ethical challenges. Regulatory hurdles include difficulties in classification and approval, the need for standardization of production, dose optimization and long‐term safety monitoring. Ethical considerations involve the complexity of obtaining informed consent, issues of equitable access, concerns about privacy and data security, the potential for enhancement and global collaboration. To successfully advance the dissemination of engineered exosome vaccines, collaboration among scientists, physicians, regulatory agencies and ethicists is imperative. This collective effort aims to ensure the safety and efficacy of treatments while balancing scientific development with ethical principles.

While the current landscape of research on engineered exosomes in BC immunotherapy holds great promise, future endeavours must focus on bridging the gap between preclinical investigations and clinical application. It is worth noting that establishing a standardized framework for the characterization and quality control of engineered exosomes is crucial to ensure consistency and reliability in preclinical and clinical research. However, there is currently no universally recognized quality control standard for such a framework, and this is an area that researchers need to further refine in the future. These efforts are essential to unlock the full clinical potential of engineered exosomes, thereby augmenting their value in BC immunotherapy.

## AUTHOR CONTRIBUTIONS

Jilong Cao and Fang Wei wrote the manuscript and created the figures. Gang Lv created the figures. Jilong Cao and Gang Lv participated in the revision of the manuscript. Fang Wei conceived the final approval of the version to be submitted and provided the funding. All authors read and approved the final manuscript.

## CONFLICT OF INTEREST STATEMENT

The authors declare no conflict of interest.

## FUNDING INFORMATION

Not applicable

## ETHICS STATEMENT

Not applicable.

## Data Availability

Not applicable.
